# Impact of cytomegalovirus infection on B cell differentiation and cytokine production in multiple sclerosis

**DOI:** 10.1186/s12974-020-01840-2

**Published:** 2020-05-20

**Authors:** Ana Zabalza, Andrea Vera, Elisenda Alari-Pahissa, Elvira Munteis, Antía Moreira, Jose Yélamos, Mireia Llop, Miguel López-Botet, Jose E. Martínez-Rodríguez

**Affiliations:** 1 Neurology Department, Hospital del Mar Medical Research Institute (IMIM), Passeig Marítim, 25-29, 08003 Barcelona, Spain; 2 Departament de Medicina, Universitat Autònoma de Barcelona, Barcelona, Spain; 3 University Pompeu Fabra, Barcelona, Spain; 4 Neurology Department, Althaia, Xarxa Assistencial i Universitària de Manresa, Manresa, Spain; 5 Hospital del Mar Medical Research Institute (IMIM), Barcelona, Spain; 6 Immunology laboratory, Hospital del Mar, Barcelona, Spain

**Keywords:** Multiple sclerosis, B cells, Human cytomegalovirus, Interferon-beta

## Abstract

**Background:**

Human cytomegalovirus (HCMV) infection has been recently associated with a low risk of multiple sclerosis (MS), yet the basis behind this observation remains uncertain. In this study, we aimed to determine in MS patients whether HCMV induces modifications in the peripheral B cell compartment.

**Methods:**

HCMV serostatus was determined in 73 MS patients (55 relapsing-remitting MS (RRMS); 18 progressive MS (PMS)) and 30 healthy controls, assessing their B cell immunophenotype and cytokine production (GM-CSF, IL-6, IL-10, and TNFα) by flow cytometry.

**Results:**

HCMV seropositivity in untreated MS patients (*n* = 45) was associated with reduced switched memory B cells, contrasting with an opposite effect in PMS. Expansions of transitional B cells were observed in HCMV(+) IFNβ-treated RRMS patients but not in HCMV(−) cases (*p* < 0.01), suggesting that HCMV may influence the distribution of B cell subsets modulating the effects of IFNβ. Considering the B cell functional profile, HCMV(−) PMS displayed an increased secretion of proinflammatory cytokines (IL-6, TNFα) as compared to HCMV(+) PMS and RRMS cases (*p* < 0.001).

**Conclusions:**

Our study reveals an influence of HCMV infection on the phenotype and function of B cells, promoting early differentiation stages in RRMS and reducing the proinflammatory cytokine profile in advanced MS forms, which might be related with the putative protective role of this virus in MS.

## Introduction

Multiple sclerosis (MS) is a demyelinating disease of the central nervous system classically considered to be mediated by T cells. A relevant role of B cells in MS pathogenesis is supported by a growing body of evidences, particularly the successful therapeutic results using anti-CD20 monoclonal antibodies [1, 2]. Epstein-Barr virus (EBV), the main pathogen related to MS [3], establishes a persistent infection having memory B cells as the main reservoir. In this regard, it is conceivable that efficacy of anti-CD20 therapies might depend not only on suppression of B cell functions, but also on the control of EBV infection [4]. On the other hand, human cytomegalovirus (HCMV) has been associated with a lower MS susceptibility based on seroepidemiological studies [5, 6], yet the basis for these observations remains poorly understood.

HCMV chronic infection leads to marked changes in the immune system [7], mainly characterized by the differentiation and expansion of specific T cells [8] and adaptive NK cell subsets expressing the CD94/NKG2C activating receptor [9], a phenotypic feature that has been associated with reduced MS progression [10]. In this study, we evaluated whether HCMV may have an influence on the B cell compartment in MS patients according to clinical features of the disease.

## Methods

### Samples

Blood was collected from healthy controls (HC) and MS patients fulfilling McDonald criteria 2017 at the Neurology Department, Hospital del Mar, Barcelona (Spain), excluding cases with relapses or corticosteroid treatment in the previous 30 days, severe concomitant disease, pregnancy, and disease-modifying therapies known to deplete peripheral lymphocytes or alter their trafficking. MS forms were defined as relapsing-remitting MS (RRMS) and progressive MS (PMS) [11].

### Herpesvirus serostatus

Standard serological diagnostic tests were performed to evaluate EBV-(LIASON®) and HCMV-specific antibodies (Roche®, Cobas602).

### B cell immunophenotype

**Fig. 1:**
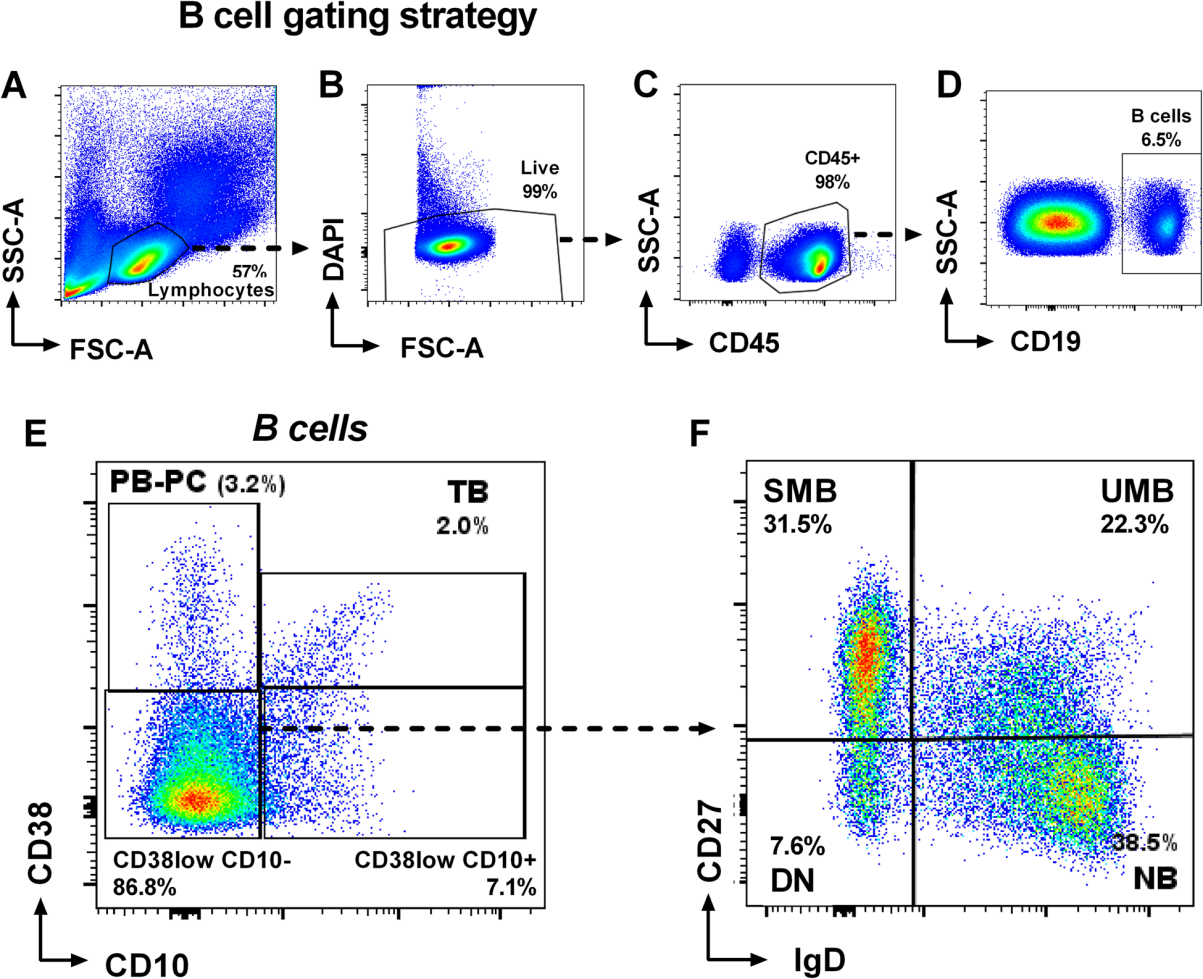
Gating strategy for B cell subset analysis. Peripheral blood mononuclear cells were gated based on forward (FSC) and side scatter (SSC) (**a**). After exclusion of DAPI(+) dead cells (**b**) and selection of CD45(+) lymphocytes (**c**), CD19+ B cell subsets (**d**) were defined as plasmablasts-plasma cells (PB-PC: CD38^hi^CD10−) and transitional (TB: CD38^hi^CD10+) (**e**), gating in the CD38^low^CD10− B cells (**f**) to define naive (NB: CD38^low/neg^CD10−IgD+CD27−), unswitched memory (UMB: CD38^low/neg^CD10−IgD+CD27+), switched memory (SMB: CD38^low/neg^CD10−IgD−CD27+), and double negative (DN: CD38^low/neg^CD10−IgD−CD27−). A representative case is illustrated in the figure, showing proportions of B cell subsets

Peripheral blood mononuclear cells (PBMCs) isolated from blood samples using Ficoll-Hypaque density gradient centrifugation were analyzed by flow cytometry using a direct staining (Fig. 1) with the following fluorochrome-conjugated monoclonal antibodies: CD19-PE-Cy7 (Invitrogen), CD38-APC, CD27-PerCP-Cy5.5 (BD Biosciences), IgD-FITC (Southern Biotech), CD10-PE (Biolegend), and DAPI (Thermo Fisher). B cell subsets were defined gating CD19+ lymphocytes as follows: plasmablasts-plasma cells (PB-PC: CD38^hi^CD10−), transitional (TB: CD38^hi^CD10+), naive (NB: CD38^low/neg^CD10−IgD + CD27−), unswitched memory (UMB: CD38^low/neg^CD10-IgD + CD27+), switched memory (SMB: CD38^low/neg^CD10−IgD−CD27+), and double negative (DN: CD38^low/neg^CD10−IgD−CD27−). Samples were analyzed in LSR II Fortessa (BD Biosciences) using FlowJo (Tree Star) software.

### Functional analysis of B cells

B cell cytokine production was evaluated in MS patients (RRMS, *n* = 45; PMS, *n* = 18) and HC (*n* = 20) incubating PBMCs for 24 h with CpG (10 μg/ml), adding in the last 4 h PMA (50 ng/mL) and ionomycin (1 μg/ml, Sigma-Aldrich), monensin (GolgiStop), and brefeldin (GolgiPlug, BD Biosciences). Samples were stained using CD19-PE-Cy7 (Invitrogen) and CD3-perCP (BD Biosciences), permeabilized, fixed, and stained with the following cytokine-specific monoclonal antibodies: anti-GM-CSF-APC, anti-IL-6-FITC (Biolegend), anti-IL-10-PE (BD Biosciences), and anti-TNFα conjugated with the CFP fluorochrome. Samples were acquired in LSR II Fortessa and data analyzed by FlowJo software, defining the proportions of B cells stained for specific cytokines.

### Statistical analysis

Kolmogorov-Smirnov test assessed normal distribution, providing means and standard deviations for parametric variables, and medians and interquartile range for non-parametric variables. Values were compared using Student’s *t* test for parametric variables or Mann-Whitney *U* test for non-parametric, respectively. Pearson or Spearman correlation indexes were calculated for pair-wise continuous variables. Results were considered significant at the two-sided level of 0.05. Data analysis was performed using GraphPad Prism 6.0 software (La Jolla, CA).

## Results

### Demographic description

We studied 30 HC and 73 MS patients (55 RRMS, 18 PMS: 8 secondary PMS, 10 primary PMS) (Table 1). HCMV serology distribution was comparable in controls and MS patients. No HCMV-related clinical differences were perceived, other than a lower disability score in HCMV(+) PMS as compared to HCMV(−) PMS patients (MSSS, 5.09 ± 2.23 vs.7.48 ± 1.28, *p* < 0.05).

**Table 1:** Demographic and clinical characteristics of MS patients and controls

		HC (*n* = 30)	RRMS (*n* = 55)	PMS (*n* = 18)	*p* value, HC vs MS	*p* value, RRMS vs PMS	*p* value, RRMS vs HC
Age, years (mean ± SD)		41.1 ± 11.4	44.7 ± 12.1	60.1 ± 8.0	**< 0.01**	**< 0.01**	0.190
Female sex, *n* (%)		18 (60.0%)	31 (56.40%)	11 (61.10%)	0.498	0.914	0.173
HCMV(+) serology, *n* (%)		23 (76.7%)	35 (63.6%)	11 (61.1%)	0.133	0.400	0.093
EBV(+) serology, *n* (%)		24 (85.7%)	54 (98.2%)	17 (94.4%)	**< 0.05**	0.075	**< 0.05**
MS duration (years)		–	10.9 (5.5–16.0)	22.6 (13.9–33.3)	–	**< 0.01**	–
EDSS		–	2.0 (1.0–3.0)	6.0 (4.4–7.0)	–	**< 0.01**	–
MSSS		–	2.08 (1.01–4.17)	6.74 (3.74–7.46)	–	**< 0.01**	–
2y-RR		–	0.0 (0.0–1.0)	0.0 (0.0–0.0)	–	**< 0.01**	–
ARR		–	0.31 (0.17–0.57)	0.0 (0–0.13)	–	**< 0.01**	–
DMT	None	–	27	18		**< 0.01**	–
	IFNβ	–	23	0			
	GA	–	5	0			

Values are expressed as mean ± SD for parametric variables and as median (interquartile range) for nonparametric variables

*HC* healthy controls, *RRMS* relapsing-remitting MS, *PMS* progressive MS, *HCMV* human cytomegalovirus, *EBV* Epstein-Barr virus, *EDSS* expanded disability status scale, *MSSS* Multiple Sclerosis Severity Score, *2y-RR* relapse rate in the previous 2 years, *ARR* annualized relapse rate, *DMT* disease-modifying therapy, *IFNβ* interferon-β, *GA* glatiramer acetate

### Distribution of B cell subsets in untreated MS according to clinical forms and HCMV serology

**Fig. 2:**
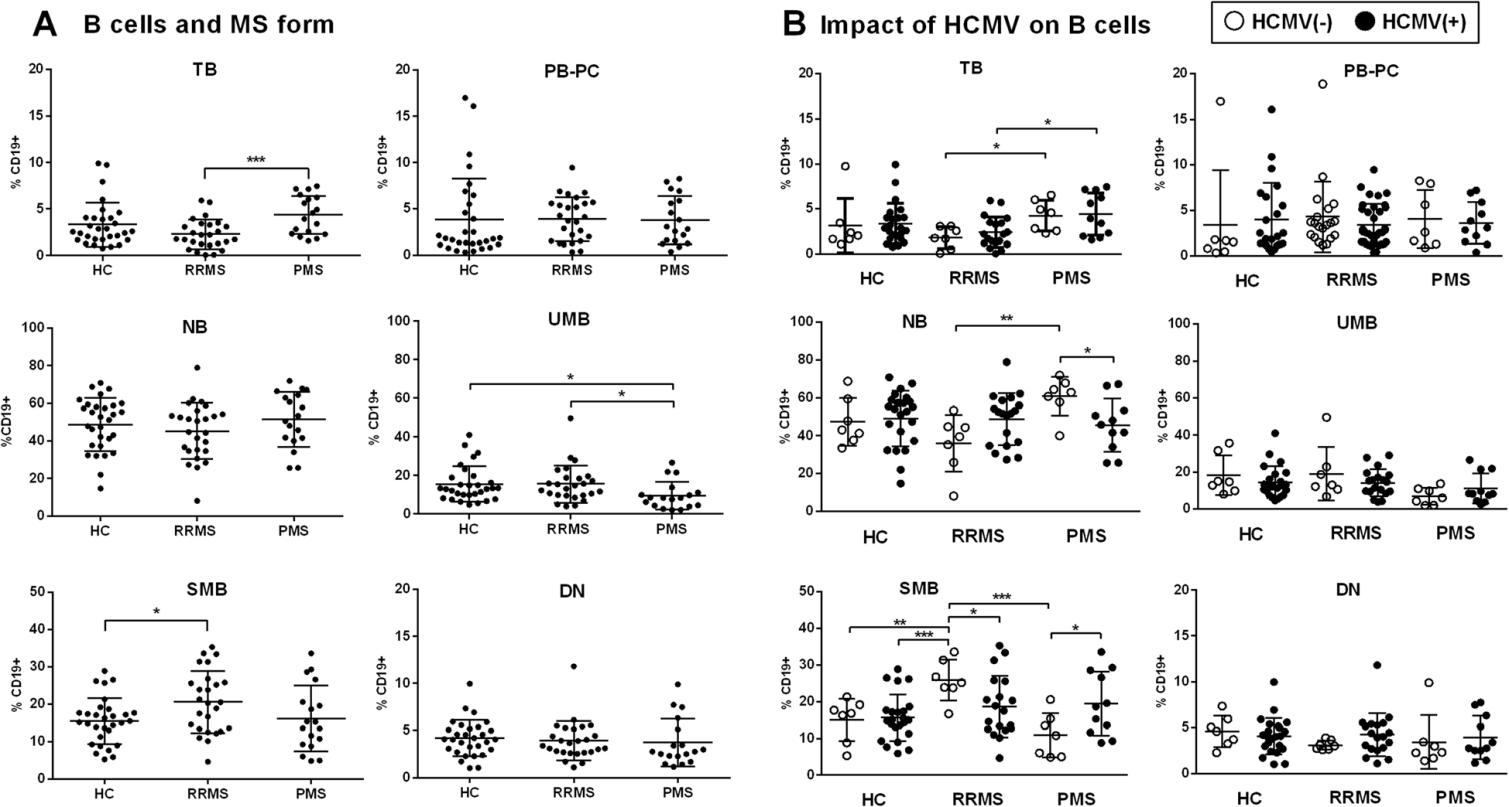
B cell subsets in controls and untreated MS patients according to clinical form and HCMV. **a** Percentages of B cell subsets within total B cells (CD19+) in healthy controls (HC) and MS patients classified based on clinical form. **b** HC and MS patients according to HCMV serostatus. RRMS: relapsing-remitting MS; PMS: progressive MS; PB-PC: plasmablasts-plasma cells; TB: transitional B cells; NB: naive B cells; UMB: unswitched memory B cells; SMB: memory switched B cells, DN: double negative B cells. *p* values: *< 0.05, ***< 0.001

Assessment of the B cell immunophenotype in MS patients without treatment (*n* = 45) and in HC did not show significant differences in the distribution of the main B cell subsets (Additional file 1: Table 1). However, stratifying MS cases according to clinical form revealed that RRMS displayed lower proportions of TB cells together with increased SMB cells as compared to HC and PMS, whereas PMS had lower proportions of UMB (Fig. 2a). Interestingly, HCMV seropositivity was associated with greater proportions of NB and reduced SMB cells in RRMS, contrasting with an opposite effect in PMS (Fig. 2b). No differences in the proportions of the different subsets were noticed in HC stratified according to HCMV serology. These results suggest that HCMV infection may differently influence the B cell compartment distribution in MS depending on the clinical form.

### Impact of HCMV on the B cell compartment in IFNβ-treated MS patients

**Fig. 3:**
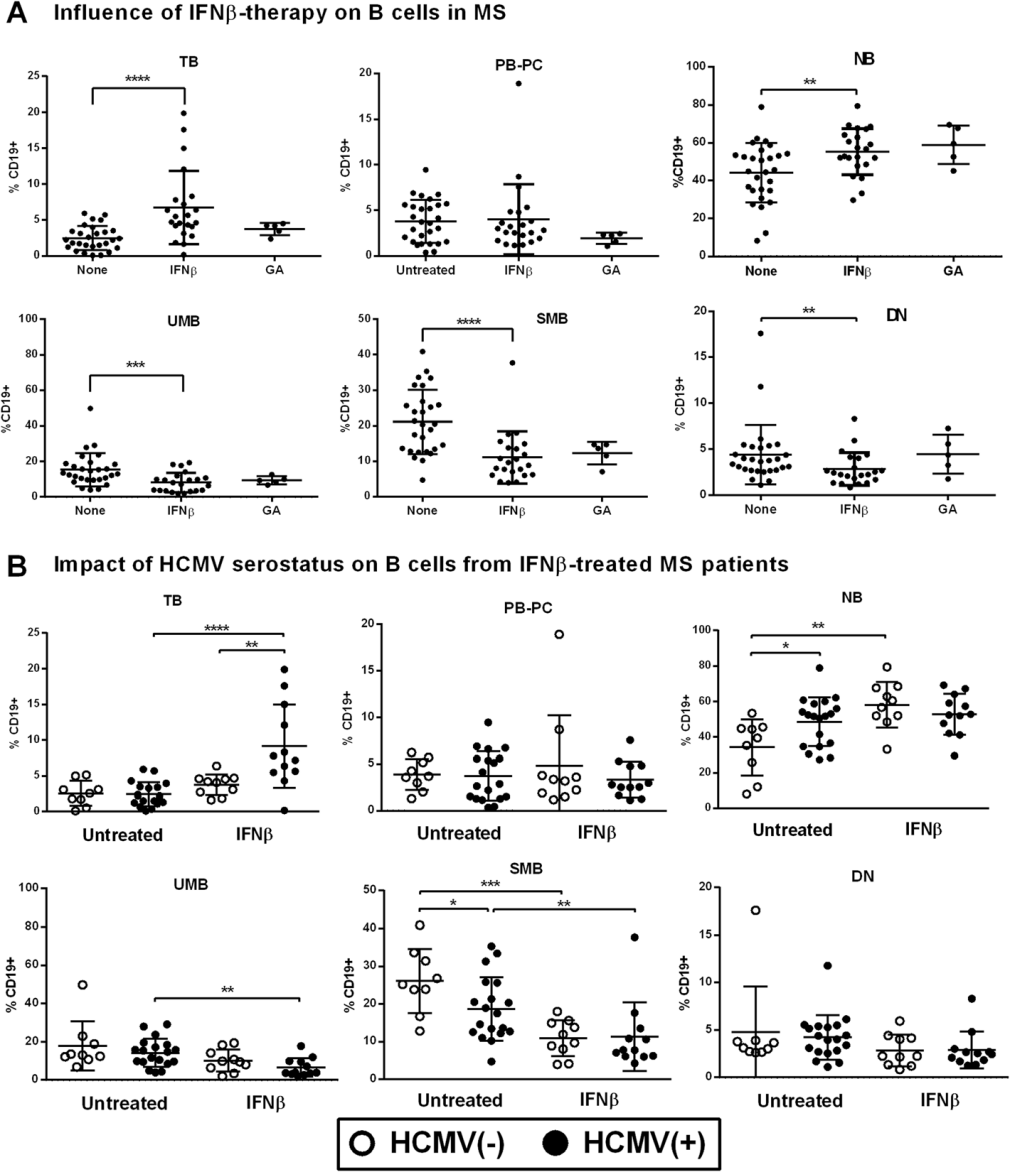
Impact of HCMV on the B cell compartment in IFNβ-treated MS patients. **a** B cell subsets in untreated (*n* = 27), IFNβ-treated (*n* = 23), and glatiramer-acetate-treated (*n* = 5) RRMS patients. **b** Untreated and IFNβ-treated MS patients classified based on HCMV serostatus. PB-PC: plasmablasts-plasma cells; TB: transitional B cells; NB: naive B cells; UMB: unswitched memory B cells; SMB: memory switched B cells; and DN: double negative B cells. *p* values: *< 0.05, **< 0.01, ***< 0.001, ****< 0.0001

IFNβ therapy in RRMS patients (*n* = 23) was associated with increased proportions of TB and NB cells, and a reciprocal reduction of UMB, SMB, and DN cells (Fig. 3a), in line with previous studies describing an effect of this cytokine on the redistribution of B cell subsets in MS patients [12]. Glatiramer acetate treatment (*n* = 5) was not associated with modifications in B cell subsets (Fig. 3a).

Remarkably, the effect of IFNβ therapy on the proportions of TB cells differed according to HCMV serostatus, observing an increase in treated HCMV(+) MS patients but not in seronegative cases (Fig. 3b). Moreover, IFNβ therapy in HCMV(−) RRMS was associated with increased NB and reduced SMB cells, resembling the effect of HCMV infection on these B cell subsets described above, which dampened the differences with HCMV(+) treated cases (Fig. 3b). These results revealed that HCMV infection may influence the distribution of B cell subsets in MS patients modulating the effects of IFNβ.

### Functional implications of HCMV on the cytokine profile of B cells in MS

**Fig. 4:**
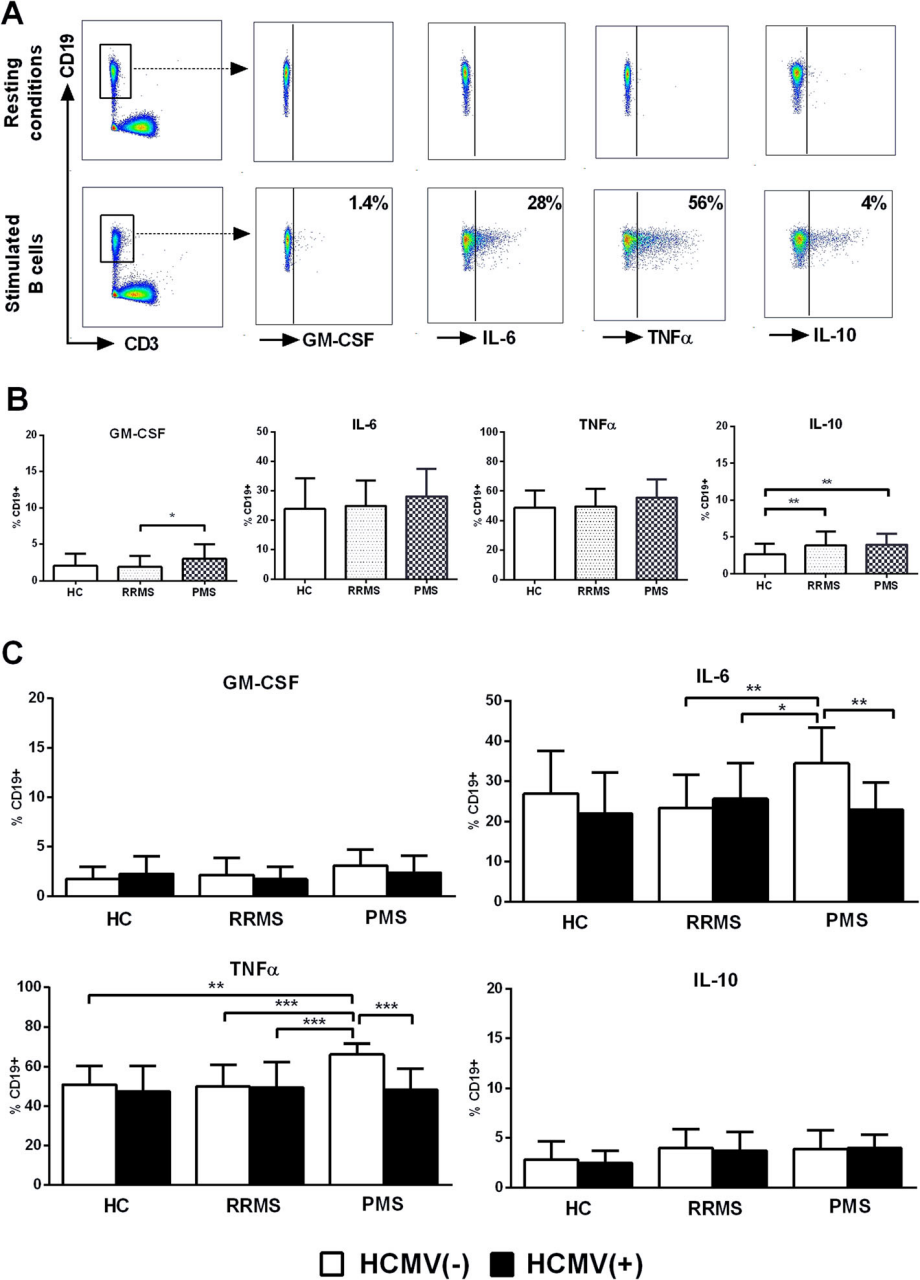
B cell cytokine production in controls and MS patients. **a** Representative cases illustrating the gating strategy and staining of pro-inflammatory (GM-CSF, IL-6, TNFα) and anti-inflammatory cytokines (IL-10) in B cells in resting conditions and stimulated using CpG and PMA/ionomycin. **b** B cell cytokine production in controls and MS patients according to MS clinical form. **c** Impact of HCMV on B cell cytokine production in controls and MS patients classified based on clinical form. *p* values: *< 0.05, **< 0.01, ***< 0.001

B cell cytokine production was evaluated as described in the “Methods” section (Fig. 4a). No differences were found between HC and the whole group of MS patients, though we observed a greater IL-10 secretion by B cells from RRMS and PMS patients as compared to HC, and increased GM-CSF by B cells from PMS as compared to RRMS patients (Fig. 4b). Considering B cell subsets, IL-10 production in MS was directly related to proportions of TB cells (*R* 0.35, *p* < 0.05) and inversely to SMB cells (*R* − 0.39, *p* < 0.05), whereas TNFα appeared inversely related to TB (*R* − 0.43, *p* < 0.01) and directly to UMB (*R* 0.49, *p* < 0.01). IFNβ therapy, age, and disability scores appeared unrelated with the B cell cytokine production (data not shown). On the other hand, HCMV(−) PMS displayed greater IL-6 and TNFα production as compared to HCMV(+) PMS and HCMV(−) RRMS patients, contrasting with comparable values of cytokine production in HCMV(+) cases (Fig. 4c). These results suggest that HCMV infection in PMS patients may decrease the production of proinflammatory cytokines by B cells.

## Discussion

In the present study, we provide data supporting that HCMV infection alters the phenotypic and function of B cells in MS, modulating the influence of IFNβ and reducing the proinflammatory B cell profile. These observations may contribute to understand the putative influence of this viral infection in MS, as suggested by previous studies describing a low risk for MS in HCMV seropositive cases [5, 6]. Although an epiphenomenon linked to the exposure to other environmental factors could not be excluded in the light of these observations, putative mechanisms of heterologous immunity between viruses [13–15] and HCMV-induced modifications on the immune system may be implicated in the effect of HCMV on MS [7, 8, 16, 17]. In this regard, HCMV has been described as an environmental factor inducing striking effects on the immune system [7]. Nevertheless, in contrast to the well-known role of HCMV as a driving force behind the expansion of late-differentiated T lymphocytes [8, 18] and adaptive NK cells [9], the influence of chronic HCMV infection on B cells is minor and barely evaluated in previous studies.

B cells have been implicated in MS pathogenesis, with a special relevance of the memory subset [2, 19]. Of note, no HCMV-related differences in B cell subsets between controls and MS patients were found in our study. However, an influence of the viral infection on the B cell subset distribution, namely the proportions of NB and SMB, was perceived when MS form was considered, with an opposite effect in RRMS as compared to PMS patients. The reduced proportions of memory B cells observed in HCMV(+) RRMS appear consistent with a protective effect of the viral infection in MS described in previous studies [5, 6]. Intriguingly, no influence of HCMV was detected in the B cell subset distribution in controls, in line with previous reports of minor HCMV-induced modifications in the B cell compartment as compared to T cells [18]. In this regard, HCMV-related differences in B cell subsets and the functional profile observed in our MS patients appeared uncoupled to markers of terminal differentiation of T cells and expansion of adaptive NKG2C+ NK cells (data not shown), suggesting specific changes on the B cell compartment induced by HCMV in the setting of MS.

Of note, the shift in the B cell subset distribution associated with HCMV in our untreated MS patients was reminiscent of the impact of IFNβ therapy in HCMV seronegative RRMS patients, an effect that might be hypothetically related with interferon-signaling pathways induced by persistent HCMV infection [20]. In addition, the previously described increase of TB cells associated with IFNβ [12] was only observed in HCMV(+) RRMS patients. In view of the implications of B cells in the mechanism of action of this cytokine [21], it is reasonable to hypothesize that HCMV infection in MS patients may modulate the influence of IFNβ on B cell differentiation.

B cells in MS have been shown to display a pro-inflammatory cytokine profile [22, 23], which was also related with MS clinical form when B cells from CSF were evaluated in PMS cases [24]. In our study, B cells from HCMV(−) PMS patients displayed an enhanced pro-inflammatory profile as compared to HCMV(+) PMS cases, independently of other clinical variables. Although limited by the reduced sample size of PMS patients evaluated, these results suggest that persistent HCMV infection may reduce the inflammatory profile of B cells in MS, in line with previous reports describing differences in IL-10 production by B cells from monozygotic healthy twins discordant for HCMV [7].

## Conclusions

Altogether, our study supports that HCMV infection modulates the distribution of B cell subsets and the IFNβ response in MS patients, and associated with a reduced pro-inflammatory cytokine profile in PMS, thus providing mechanistic insights on the putative beneficial influence of HCMV in MS. Further studies are required to assess whether HCMV may also modulate the immunological response to other MS therapies targeting B cells [19, 25].

## Supplementary information

**Additional file 1: Table 1.** Proportions of B cell subsets in controls and MS patients according to HCMV serostatus.

## Data Availability

The datasets used and/or analysed during the current study are available from the corresponding author on reasonable request.

## Abbreviations

EBV: Epstein-Barr virus
HCMV: Human cytomegalovirus
IFNβ: Interferon-beta
MS: Multiple sclerosis
PBMCs: Peripheral blood mononuclear cells
PMS: Progressive multiple sclerosis
RRMS: Relapsing-remitting multiple sclerosis
