# Supplementary material for: Impact of cytomegalovirus infection on B cell differentiation and cytokine production in multiple sclerosis
Source: J Neuroinflammation. 2020 May 20;17:161. doi: 10.1186/s12974-020-01840-2 (PMC7238600; doi:10.1186/s12974-020-01840-2)
Supplement: Supplementary file 1 — Additional file 1: Table 1. Proportions of B cell subsets in controls and MS patients according to HCMV serostatus. [file 12974_2020_1840_MOESM1_ESM.docx]

**Additional Table 1. Proportions of B cell subsets in controls and MS patients according to HCMV serostatus**.

|  |  | **Controls** |  |  | **MS patients** |  |  |  |
| --- | --- | --- | --- | --- | --- | --- | --- | --- |
|  | **HCMV(-) (n=7)** | **HCMV(+) (n=23)** | **P-value** | **HCMV(-) (n=27)** | **HCMV(+) (n=46)** | **P-value** | **P-value #** | **P-value §** |
| **TB** | 3.1 ± 3.0 | 3.4 ± 2.2 | 0.335 | 3.5 ± 1.7 | 4.8 ± 4.3 | 0.591 | 0.677 | 0.291 |
| **PB-PC** | 3.4 ± 6.0 | 4.0 ± 4.0 | 0.207 | 4.2 ± 3.7 | 3.4 ± 2.3 | 0.395 | **<0.05** | 0.693 |
| **NB** | 47.4 ± 12.5 | 49.2 ± 14.7 | 0.768 | 50.7 ± 17.3 | 50.0 ± 13.3 | 0.851 | 0.527 | 0.894 |
| **UMB** | 18.3 ± 10.8 | 14.5 ± 8.7 | 0.360 | 11.9 ± 9.3 | 11.1 ± 7.2 | 0.656 | 0.378 | 0.058 |
| **SMB** | 15.1 ± 5.8 | 15.7 ± 6.3 | 0.819 | 16.1 ± 9.5 | 16.3 ± 8.9 | 0.945 | 0.787 | 0.789 |
| **DN** | 4.6 ± 1.7 | 4.1 ± 2.0 | 0.537 | 3.8 ± 3.4 | 3.8 ± 2.2 | 0.497 | 0.211 | 0.568 |

Values are expressed as mean ± standard deviation. # P-value comparing HCMV(-) controls and MS patients. § P-value comparing HCMV(+) controls and MS patients. TB: transitional B cells. PB-PC: plasmablasts / plasmatic cells. NB: naïve B cells. UMB: unswitched memory B cells. SMB: memory switched B cells. DN: double negative.
